# Editorial: Theoretical Syntax at the Crossroads: Big Data, Citizen Science and Crowdsourcing

**DOI:** 10.3389/fpsyg.2021.755889

**Published:** 2021-10-05

**Authors:** Ángel J. Gallego, Ivan Ortega-Santos

**Affiliations:** ^1^Department of Spanish Philology, Autonomous University of Barcelona, Barcelona, Spain; ^2^Department of World Languages and Literatures, University of Memphis, Memphis, Memphis, TN, United States

**Keywords:** linguistic theory, syntax, big data, crowdsourcing, theory, data, citizen science, methodology

The study of theoretical syntax has experienced a series of changes over the last decades, progressively incorporating new data gathering techniques that had both a methodological and conceptual impact. Under the labels “big data” and “citizen science,” one can find strategies to cover more empirical terrain and, at the same time, reconsider the data-theory balance. Within this relatively broad background, the goal of this Research Topic is to provide an overview of the opportunities for innovation and the challenges inherent in the emerging paradigm to help researchers navigate and take advantage of these changes. In particular, we welcomed methodological contributions and detailed case studies on specific languages that explore the following issues (as well as others):

## Big Data: How to Make Use of These Resources Fruitfully and What Are Their Limits?

The use of big data for theoretical linguistics provides a unique access to subpopulations and linguistic phenomena under-represented in the theoretical literature. At the same time, there are potential granularity mismatches in data sets studied within theoretical syntax, which may include highly infrequent, even unattested or ungrammatical data while controlling for independent factors, and those available through the study of big data.

## Crowdsourcing and Citizen Science: How to Make Use of These Resources While Controlling for Data Quality and Avoiding Potential Ethical Issues?

The use of these resources entails analyzing data from untrained informants. This may help avoid potential shortcomings like experimental biases, but potential data quality issues call for extensive validation studies as well as research on strategies to improve data quality. Furthermore, ethical concerns may arise in the use of popular crowdsourcing services as Amazon's Mechanical Turk or in the case of citizen science.

## Statistics Vs. Traditional Theoretical Research: How Can Theoretical Syntax Benefit From This Paradigm Change?

The link between big data, citizen science and crowdsourcing, on the one hand, and statistical scrutiny on the other, favors a debate on the advantages and limits of the use of statistical tools standard in Cognitive Science for the study of Syntax, including but not limited to issues relating to the convergence rate between data collected using traditional methods and data gathered as part of the said paradigm change. Likewise, the use of big data, crowdsourcing and citizen science provides an opportunity to tap into varieties and phenomena that might not even be present in current corpora or the theoretical literature, thus opening the door to the fruitful study of microvariation combined with statistics (dialectometry) to increase the empirical basis of the studies on microvariation.

The present contributions take up these questions emphasizing: (i) the importance of crowdsourcing in the study of non-standardized varieties, which reveal a non-trivial amount of variation thus calling for experimental data gathering and statistical analysis; (ii) the opportunities that corpora and other resources provide to ask questions that we could not tackle before; and (iii) the relationship between the nature of syntactic theory and the relevance of the availability of massive amounts of data.

Sheehan et al. focus on the challenges and opportunities of the use of crowdsourcing for the analysis of minority languages through a case study on Galician and its import for so-called Control Theory within Generative Grammar. Specifically, they provide an argument in favor of a variety of factors such as the use of crowdsourcing to gather data efficiently, the inclusion of sociolinguistic variables to avoid privileging a specific sociolect, and the use of statistics and experimental methodology, particularly for cases where gradience in the perception of acceptability syntactic data is attested.

The challenges of gathering data of minority languages are also present in Leivada et al.. According to these scholars, doing research on small, young or non-standard varieties is challenging because of a number of issues, such as inter- and intraspeaker variation, potentially due, among other things, to the lack of standardization, absence of corpora of naturalistic speech, absence of information on features relevant for experimental design (e.g., word frequency in the case of acceptability judgment tasks) or absence of orthographic conventions or theoretical descriptions. Solutions are provided when possible. For instance, it is suggested that big(ger) participant samples even for relatively small populations and the combination of a variety of methodologies (e.g., acceptability judgment tasks with corpora search or group discussions among the participants) are crucial to achieve an accurate description of these kinds of varieties.

The relationship between statistics and theory is taken up by Cognola et al.. Specifically, they provide evidence for the relevance of quantitative research for the study of microvariation, in particular, heritage varieties, which show inter- and intraspeaker variation. This methodology is crucial to allow the researchers to obtain reliable data from Mòcheno, a German minority heritage language from Italy, in spite of the variation attested in the community.

de Toledo y Huerta discusses the wealth of data available in corpora, digital libraries and/or the internet for the study of syntactic change and the new opportunities these provide. In particular, these resources have made it possible for researchers to focus on low frequency phenomena and on evolutionary curves of linguistic phenomena (as opposed to specific periods), setting the stage for the novel study of linguistic diffusion.

Finally, Mendívil-Giró explores how much of an influence big data may have on syntactic theorizing. Deductive approaches as Generative Grammar and inductive ones as Functionalism both value data—as necessary to be falsifiable. Still, the former is theory-driven and arguably can reach generalizations from the study of a single language, though crosslinguistic work is relevant as well. In contrast, the latter focuses on data primarily to arrive at generalizations. Thus, big data is arguably more likely to have an effect on functionalist approaches than generative ones.

In addition to such fruitful (and necessary) discussion, the scope of the contributions underscores the fluid relationship between linguistics and so-called Digital Humanities, traditionally seen in corpora, online linguistic atlases, and similar resources.

## Author Contributions

All authors listed have made a substantial, direct and intellectual contribution to the work, and approved it for publication.

## Funding

This research has been partially supported by two grants from the Ministerio de Economía y Competitividad (FFI2017-87140-C4-1-P) and the Generalitat de Catalunya (2017SGR634). Usual disclaimers apply.

## Conflict of Interest

The authors declare that the research was conducted in the absence of any commercial or financial relationships that could be construed as a potential conflict of interest.

## Publisher's Note

All claims expressed in this article are solely those of the authors and do not necessarily represent those of their affiliated organizations, or those of the publisher, the editors and the reviewers. Any product that may be evaluated in this article, or claim that may be made by its manufacturer, is not guaranteed or endorsed by the publisher.

